# Assessment of patients’ self-perceived intensive care unit discomforts: Validation of the 18-item version of the IPREA

**DOI:** 10.1186/s12955-019-1101-5

**Published:** 2019-02-07

**Authors:** Karine Baumstarck, Mohamed Boucekine, Philippe Estagnasie, Marie-Agnès Geantot, Audrey Berric, Georges Simon, Bernard Floccard, Thomas Signouret, Mélanie Fromentin, Martine Nyunga, Achille Sossou, Marion Venot, René Robert, Arnaud Follin, Juliette Audibert, Anne Renault, Maïté Garrouste-Orgeas, Olivier Collange, Quentin Levrat, Isabelle Villard, Didier Thevenin, Julien Pottecher, René-Gilles Patrigeon, Nathalie Revel, Coralie Vigne, Elie Azoulay, Olivier Mimoz, Pascal Auquier, Pierre Kalfon, the IPREA Study group, Karine Vie, Gwenaëlle Lannuzel, Hélène Bout, Jean-Philippe Parthiot, Isabelle Chazal, Philippe Charve, Caroline Prum, Jean-Pierre Quenot, Nora Perrot, Francis Augier, Niloufar Behechti, Claudine Cocusse, Céline Foulon, Laurence Goncalves, Abdesselem Hanchi, Etienne Legros, Ana Isabel Mercier, Nicolas Meunier-Beillard, Nathalie Nuzillat, Alicia Richard, Claire Boulle, Benjamin Kowalski, Elisa Klusek, Tarek Sharshar, Andrea Polito, Caroline Duvallet, Sonia Krim, Nicolas Girard, Juliette Audibert-Souhaid, Cécile Jourdain, Stéphane Techer, Corinne Chauvel, Corinne Bruchet, Johanna Temime, Stéphanie Beaussart, Fabienne Jarosz, Julien Crozon-Clauzel, Serge Olousouzian, Sylvie Pereira, Loïc Argentin, Valérie Cerro, Déborah Levy, Sébastien Andre, Christophe Guervilly, Laurent Papazian, Myriam Moussa, Stéphanie Renoult, Delphine Biet, Steve Novak, Jean-Christophe Orban, Aminata Diop, Carole Ichai, Antoine Tesniere, Jean-Pascal Goupil, Frédérique Laville, Nadège Rutter, Sandie Brochon, Kelly Tiercelet, Julien Amour, Nora Ait-Hamou, Marjorie Leger, Virginie Souppart, Emilie Griffault, Marie-Line Debarre, Céline Deletage, Anne-Laure Guerin, Carole Guignon, Sabrina Seguin, Christophe Hart, Kathy Dernivoix, Caroline Wuiot, Karine Sanches, Stéphane Hecketsweiler, Catherine Sylvestre-Marconville, Vincent Gardan, Stéphanie Deparis-Dusautois, Yana Chaban

**Affiliations:** 1 Aix-Marseille Univ, School of medicine - La Timone Medical Campus, EA 3279 CEReSS - Health Service Research and Quality of Life Center, |27 bd Jean Moulin cedex 05, F-13385 Marseille, France; 2 Réanimation, Clinique Ambroise Paré, Neuilly/Seine, France; 3 Département d’Anesthésie Réanimation, CHU Dijon Bourgogne, Dijon, France; 4 Réanimation polyvalente, Centre Hospitalier Intercommunal Toulon/La Seyne sur mer, Toulon, France; 5 Réanimation, CH Troyes, Troyes, France; 6 Réanimation polyvalente, CHU Edouard Herriot, Hospices Civils de Lyon, Lyon, France; 7 Réanimation, Hôpital Européen de Marseille, Marseille, France; 8 Réanimation chirurgicale, CHU Cochin, Assistance Publique-Hôpitaux de Paris (AP-HP), Paris, France; 9 Réanimation polyvalente, CH Victor Provo, Roubaix, France; 10 Réanimation, CH Emile Roux, Le Puy-en-Velay, France; 11 Réanimation médicale, CHU Saint-Louis, AP-HP, Paris, France; 12 Réanimation médicale, CHU La Milétrie, Poitiers, France; 13 Réanimation chirurgicale, Hôpital Européen Georges Pompidou, AP-HP, Paris, France; 14 Réanimation polyvalente, Hôpital Louis Pasteur, CH de Chartres, Le Coudray, France; 15 Réanimation médicale, CHU Brest, Brest, France; 16 Médecine intensive et réanimation, Groupe Hospitalier Paris Saint-Joseph, Paris, France; 17 Réanimation chirurgicale polyvalente, Hôpital Civil, CHU Strasbourg, Strasbourg, France; 18 Réanimation, Groupe Hospitalier de La Rochelle-Ré-Aunis, La Rochelle, France; 19 Anesthésie Réanimation, CHU Beaujon, AP-HP, Clichy, France; 20 Réanimation, CH Lens, Lens, France; 21 Réanimation chirurgicale, Hôpital Hautepierre, CHU Strasbourg, Strasbourg, France; 22 Réanimation, CH Auxerre, Auxerre, France; 23 Réanimation médico-chirurgicale, Hôpital Pasteur, CHU Nice, Nice, France; 24 Réanimation chirurgicale, CHU Hôpital Nord, Assistance Publique–Hôpitaux de Marseille, Marseille, France

**Keywords:** IPREA, Discomfort, Critical care, Validation, Questionnaire

## Abstract

**Background and aims:**

We reported the validation of the 18-item version of the ‘Inconforts des Patients de REAnimation (IPREA)’ questionnaire that includes 2 new items exploring feeling depressed and shortness of breath during an intensive care unit (ICU) stay.

**Methods:**

The validation process was integrated in a multicenter, cluster-randomized, controlled, two-parallel group study built to assess the effectiveness of a tailored multicomponent program for reducing self-perceived discomfort in the ICU. All patients aged 18 years or older who survived an ICU stay of 3 calendar days or more were eligible for inclusion. Data collection included demographics (sex, age), type of admission (medical and surgical), health status scores at admission (Knaus score and McCabe index, Simplified Acute Physiology Score (SAPS) II), specific ICU therapeutics such as mechanical ventilation (MV), noninvasive ventilation (NIV), use of vasopressors, or renal replacement therapy (RRT), and ICU stay duration.

**Results:**

A total of 994 patients were included. The initial structure of IPREA was confirmed using confirmatory factor analysis showing satisfactory fit (RMSEA at 0.042, CFI at 0.912). No multidimensional structure was identified, allowing the calculation of an overall discomfort score. The three highest discomforts were sleep deprivation, thirst, and perfusion lines and other devices, and the 3 lowest discomforts were limited visiting hours, hunger, and isolation. The overall discomfort score of the 18-item version of IPREA did not differ between men and women. Higher age was significantly correlated with a lower overall discomfort score. While MV was not linked to self-reported discomfort, patients treated by NIV reported higher overall discomfort scores than patients not treated by NIV.

**Conclusion:**

The 18-item version of IPREA is easy to use and possesses satisfactory psychometric properties. The availability of a reliable and valid French questionnaire asking about patients’ self-perceived ICU discomforts enables feedback from the health care team to be incorporated in a continuous quality health care improvement strategy.

**Trial registration:**

clinicaltrial.gov NCT02442934 (registration date: May 18, 2015, retrospectively registered).

## Background

Critically ill patients experience various discomforts during their intensive care unit (ICU) stay. These discomforts may traditionally be distinguished as discomforts related to the environment (noise, light, temperature, etc.), discomforts related to some aspects of care organization (continuous monitoring, limited visiting hours, privacy not guaranteed, etc.), and discomforts related to specific ICU therapeutics such as mechanical ventilation (MV), noninvasive ventilation (NIV), renal replacement therapy (RRT), or painful procedures [1]. Recognizing these sources of discomfort in the ICU is a first step for optimizing patient comfort in the ICU through a tailored program aimed to identify and quantify discomfort sources, understand reasons for them, initiate care strategies to prevent, remove or reduce them, and assess potential improvements due to such programs in the health status of survivors of critical illness after an ICU stay.

Detection of discomforts may be performed using objective measures characterizing some stressors, such noise or excess lighting, or by measuring the impact of these stressors on physiologic parameters, or through subjective measures, including patient-reported measures. Patient-reported outcomes are now recognized as a satisfactory picture of patient perceptions that has led to the development of specific ICU-related perceived discomfort tools. From the available tools [2, 3], the ‘Inconforts des Patients de REAnimation (IPREA)’ questionnaire [4], as a self-perceived ICU discomfort measure, is differentiated by a validation process based on international guidelines, performed using a large sample of patients managed in various types of ICUs (medical and surgical). The IPREA questionnaire is a 16-item self-administered questionnaire with satisfactory psychometric properties and good acceptability that makes it relevant for implementation in routine clinical practice. According to the item selection step, some discomforts were not retained in the final version of the questionnaire. The clinical use of IPREA and an update of the literature review [5, 6] highlighted the recurrent dissatisfaction of both ICU healthcare workers and ICU patients with two main missing items, concerning ICU-related mood disorders and ICU-related breathing discomfort: feeling depressed and shortness of breath occurring during the ICU stay.

Dyspnea is prevalent in mechanically ventilated patients [7] but also in patients experiencing VNI [8]. Often underestimated by caregivers, dyspnea or shortness of breath as perceived by the ICU patients may expose them to anxiety and fear and consequently complicate care. Authors have previously emphasized the need for further development and standardization of methods to assess dyspnea in ICU patients [9].

Survivors of critical illness have high rates of depression and post-traumatic stress disorder, and depression has been found to be independently associated with an increased risk for rehospitalization [10–12]. Few studies have explored the phenomenon specifically during the ICU stay, although the clinically apparent symptoms of depression and anxiety are often present before the ICU discharge and commonly acknowledged by health care workers and caregivers [13]. The presence of these symptoms during the ICU stay has also been shown to be one of the strongest risk factors for poor psychological outcomes after critical care. Based on these findings, international health agencies [14] announced that patients should be assessed during their critical care stay for detecting and recognizing psychological stress using practical routine clinical tools [15].

The IPREA study group also proposed adding two items to the initial version of the IPREA questionnaire, leading to an 18-item version. We reported the metric validity of the 18-item version of the IPREA questionnaire including 2 new items exploring ICU-related feelings of depression and ICU-related breathing discomfort.

## Methods

### Sample and design

**Fig. 1:**
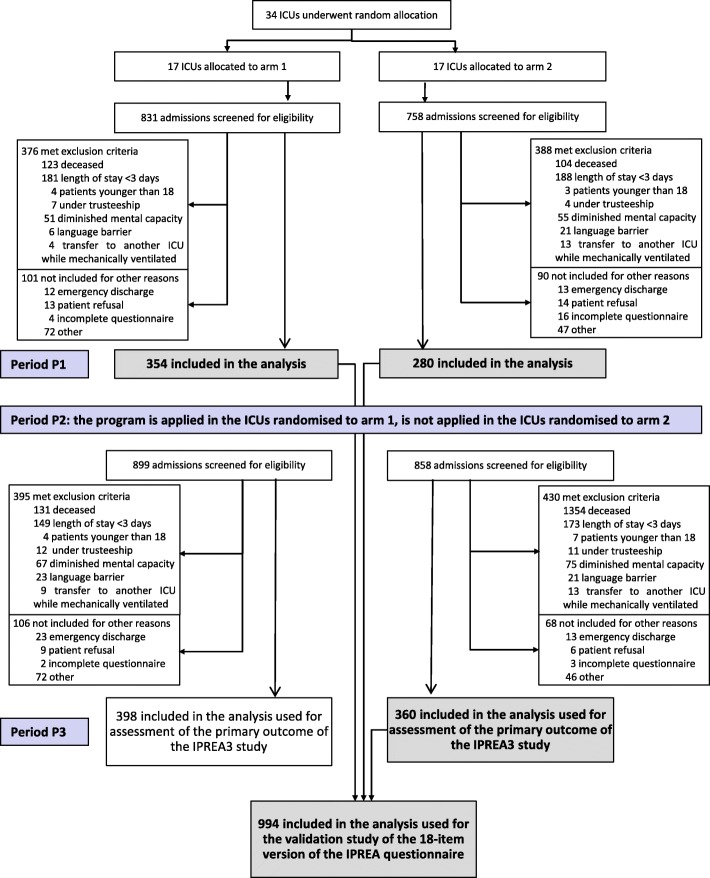
Chart

The participants of this validation step were patients included in a multicenter, cluster-randomized, controlled, two-parallel group study built to assess the effectiveness of a tailored multicomponent program (TMCP) for reducing self-perceived discomfort in the ICU, previously detailed [16, 17]. In this study, 34 French ICUs, that were medical, surgical, or mixed medical-surgical ICUs located at academic tertiary care hospitals or community hospitals, were randomized to either an experimental arm during which the TMCP (identification of discomforts, immediate feedback to the healthcare team, and implementation of targeted interventions) was implemented or to a control arm during which any program was implemented. The TMCP (described elsewhere [16, 17]) targeting all members of the healthcare team consisted of discomfort assessment with IPREA, immediate feedback to bedside nurses and monthly feedback to the healthcare team, and tailored site-targeted interventions. For the validation of the 18-item version of IPREA, only patients admitted to the ICU in which any program was implemented (Fig. 1) were eligible.

All patients aged 18 years or older who survived an ICU stay of 3 calendar days or more were eligible for inclusion. We excluded patients who died during the ICU stay, patients under trusteeship, patients with diminished mental capacity (patients with delirium were excluded), patients who did not understand French sufficiently to be questioned, and patients transferred to another ICU while mechanically ventilated.

### Ethics, consent and permissions

Regulatory monitoring was performed in accordance with the French law requiring the approval of the French ethics committee (Comité de Protection des Personnes Tours Région Centre-Ouest 1, 28/08/2013, reference number 2013-S10). All records and subject’ identities remained confidential in accordance with French regulations: the French National Committee of Informatics and Liberties (Commission nationale de l’informatique et des libertés, 20/03/2014, reference number DR-2014-097) and the French Consultative Committee for the data processing in health research (Comité consultatif sur le traitement de l’information en matière de recherche dans le domaine de la santé, 12/12/2013, reference number 13.642bis). Consent was obtained from each participant.

### Procedure and data collection

Each participating ICU was supplied with tablets with Internet connection. On the day of ICU discharge, if the patient presented eligibility criteria and had no exclusion criteria, the bedside nurse was to administer the self-perceived ICU discomfort questionnaire. To ensure adequate training of the nursing staff, the application had been in use in each ICU for a training period with technical and educational support provided by the coordination team.

Data related to the patient were recorded from an electronic case report form. The following data were collected: demographics (sex, age), type of admission (medical, scheduled surgical, and emergency surgical), health status before the ICU stay using the Knaus score and the McCabe index, health status at ICU admission using the Simplified Acute Physiology Score (SAPS II), health status from the ICU admission to ICU discharge (mechanical ventilation, noninvasive ventilation, administration of vasopressors, and renal replacement therapy), and duration of ICU stay.

The patients’ discomfort was assessed using the French self-reported discomfort IPREA questionnaire (‘Inconforts des Patients de REAnimation’, Discomforts perceived by ICU patients), including 18 discomfort-items: noise, excess of light, discomfort related to sleeping in a different bed from home, sleep deprivation, thirst, hunger, feeling of cold, feeling of heat, pain, being tied down by perfusion lines, tubes or other connected monitoring devices, no respect for privacy, anxiety, isolation, limited visiting hours, absence of phone, lack of information, shortness of breath, and felling depressed. Each item was scored from 0 (minimal discomfort) to 10 (maximal discomfort), yielding 18 linearized scores and an overall score of discomfort scored from 0 (minimal discomfort) to 100 (maximal discomfort). The questionnaire was administered on the day of ICU discharge. The timeframe considered the period from date of admission to the ICU until the day of discharge from the ICU.

### Statistical analysis

The dimensional structure of the 18-item questionnaire was performed using a confirmatory factor analysis (CFA) using the Mplus software package [18]. The fit to the model was tested by computing the root mean square error of approximation (RMSEA) and comparative fit index (CFI). RMSEA is acceptable if < 0.08 and satisfactory if < 0.05, and CFI is acceptable if > 0.9 [19, 20]. Means and standard deviations were reported for each item. Floor and ceiling effects were reported assessing the homogeneous repartition of the response distribution. For each dimension, internal consistency reliability was assessed using Cronbach’s alpha coefficient. A Cronbach’s alpha coefficient of at least 0.7 was expected for each scale [21, 22]. The unidimensionality of each scale was assessed using Rasch analyses: item goodness-of-fit statistics (INFIT) and coefficient of Loevinger (H). INFIT statistics ranging between 0.7 and 1.2 and an H coefficient of at least 0.40 ensure that all the items of the scale tend to measure the same concept [23]. Uniform and non-uniform differential item functioning (DIF) analyses following Crane’s procedure [24] were performed to compare the differences in item difficulties between groups (sex, Knaus score, and the McCabe index). The discriminant validity was determined by assessing the associations between the IPREA scores and sociodemographic and clinical features. For qualitative variables, the mean dimension scores of the IPREA were compared across patient groups that were expected to differ (e.g., sex, Knaus chronic health status [25], MacCabe classification [26], type of admission, MV, NIV, use of vasopressors, and RRT) using Student’s t test. Quantitative variables (e.g., age, SAPS II, duration of ICU stay) were analyzed using Pearson’s correlation coefficients. The underlying assumptions were derived from the initial validation of IPREA [4]: women should report higher perceived discomforts than men, older patients should report lower discomforts, more severe patients (SAPS II) should report higher discomforts, and a patient’s ICU-stay duration should be correlated to discomfort levels. For informational purpose, the same procedure was replicated on the 16 items included in the first validation [4]. Data analyses were performed using R software and Stata 9.0. software.

## Results

### Sample characteristics

From the 34 French ICUs, 2447 patients were eligible, and the study sample included 994 patients resulting to a ratio of included/eligible patients of 41%. Because of incomplete questionnaires, 23 patients were not included in the analysis. Reasons for non-inclusion were detailed. All the details are provided in Fig. 1. Key clinical and demographic characteristics are provided in Table 1.

**Table 1:** Sample characteristics

		*N* = 994
At admission		N (%)
Sex	Men	643 (64.7)
	Women	351 (35.3)
Age (years)	M (SD)	63.2 (15.6)
Type of patients	Medical	486 (48.9)
	Scheduled surgical	308 (31.0)
	Emergency surgical	200 (20.1)
Knaus score	Normal health status	228 (22.9)
	Moderate/severe limitation	766 (77.1)
MacCabe score	Non-fatal disease	597 (60.1)
	Ultimately/rapidly fatal disease	397 (39.9)
SAPS II	M (SD) m [IQR]	35.6 (16.6) 33 [23–46]
During ICU stay		
Mechanical ventilation		531 (53.4)
Noninvasive ventilation		333 (33.5)
Vasopressors administration		388 (39.0)
Renal replacement therapy		85 (8.6)
ICU stay duration (days)	M (SD) m [IQR]	7,8 (9,6) 5 [3–8]

*M (SD)* Mean (standard deviation), *m [IQR]* Median [interquartile range]

SAPS II Simplified Acute Physiology Score II (range from 0 to 156, with higher scores indicating more severe illness)

### Construct validity and internal structural validity

**Fig. 2:**
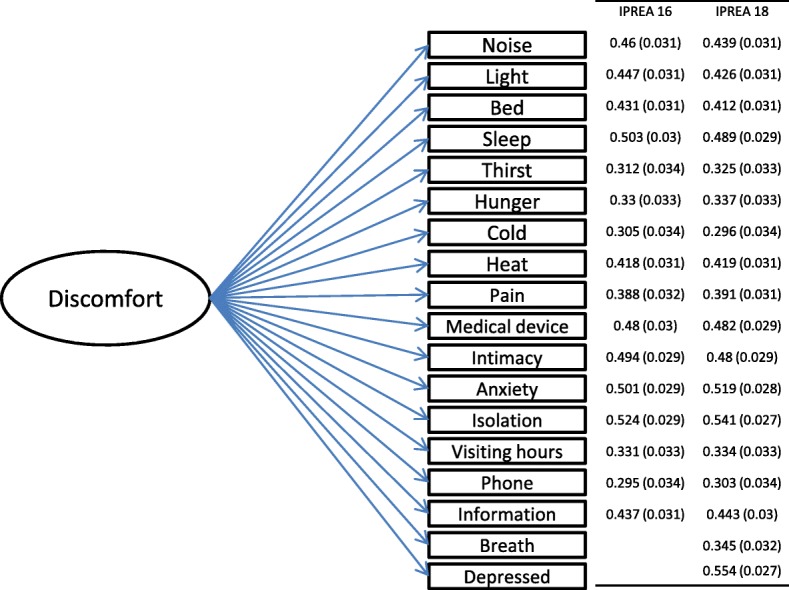
Confirmatory factor analysis

The structure was confirmed using CFA, which showed a satisfactory fit (RMSEA at 0.042, CFI at 0.912). No multidimensional structure was identified, allowing the calculation of an overall score. The component factor analysis is illustrated in Fig. 2. The three highest discomforts were sleep deprivation, thirst, and perfusion lines/devices, and the 3 lowest discomforts were limited visiting hours, hunger, and isolation.

Ceiling effects were considered satisfactory (lower than 10%), but floor effects were high for all the items. Internal consistency was satisfactory (Cronbach’s alpha: 0.80). Eight dimensions showed a satisfactory scalability. All the items showed an INFIT statistics inside the acceptable ranges. All results are provided in Table 2. Data from the 16-item version are provided in Table 2 as informative findings.

**Table 2:** Dimensions’ characteristics of the 18-item IPREA

Item	Label	M ± SD ^a^	Floor effect %	Ceiling effect %	Cronbach’s alpha		INFIT	
			18i version	18i version	18i version	*16i version*	18i version	*16i version*
1	Noise	29.58 ± 31.73	40.64	4.33	0.784	*0.757*	0.96	*0.93*
2	Excess of light	18.91 ± 27.93	58.25	2.31	0.785	*0.759*	0.95	*0.92*
3	Different bed	24.81 ± 30.75	48.69	3.72	0.790	*0.765*	1.04	*1.02*
4	Sleep deprivation	38.5 ± 33.97	32.8	5.63	0.782	*0.755*	0.92	*0.9*
5	Thirst	31 ± 35.04	45.67	7.24	0.794	*0.773*	1.14	*1.15*
6	Hunger	13.54 ± 26	71.33	2.62	0.792	*0.769*	1.05	*1.05*
7	Feeling of cold	18.88 ± 28.34	60.36	2.31	0.796	*0.773*	1.16	*1.14*
8	Feeling of heat	14.29 ± 26.59	70.32	1.91	0.790	*0.767*	1.03	*1.04*
9	Pain	29.75 ± 29.49	36.22	2.31	0.790	*0.767*	1.05	*1.05*
10	Perf. lines/devices	33.36 ± 31.75	34.61	4.43	0.785	*0.761*	0.98	*0.98*
11	Intimacy	14.15 ± 24.95	66.6	1.71	0.787	*0.761*	0.94	*0.93*
12	Anxiety	27.05 ± 31.66	46.78	3.82	0.782	*0.760*	0.92	*0.97*
13	Isolation	13.19 ± 24.2	68.91	1.21	0.784	*0.759*	0.88	*0.89*
14	Visiting hours	9.64 ± 21.71	77.77	1.41	0.793	*0.770*	1.03	*1.02*
15	Phone	14.1 ± 28.37	73.74	4.02	0.794	*0.771*	1.12	*1.11*
16	Information	20.05 ± 28.74	57.04	2.41	0.788	*0.764*	1.01	*1.01*
17	Breathing	28.42 ± 30.94	42.25	3.72	0.793		1.1	
18	Mood disorders	14.36 ± 25.73	68.21	1.91	0.781		0.85	
	*16-item total*					*0.776*		*1.01*
	18-item total^a^	21.87 ± 13.75	1.1	0	0.798		1.01	

M ± SD mean ± standard deviation; m (IQR) median (interquartile range); INFIT Rasch statistics

^a^scores ranging from 0 to 100; the higher the score, the higher the discomfort

Italic values: results of 16-item IPREA on the present sample

### External validity

The overall discomfort score of the 18-item version of IPREA did not differ between men and women. Higher age was significantly correlated with a lower discomfort score. The overall score did not differ according to Knaus and MacCabe classification. Surgical patients reported lower overall discomfort scores than did medical patients. Patients with higher SAPS II reported higher overall discomfort scores. While MV was not linked to the overall discomfort score, patients treated by NIV reported higher overall discomfort scores than patients not treated by NIV. The ICU-stay duration was positively correlated to the overall discomfort score. All details are provided in Table 3.

**Table 3:** Comparisons and correlations of IPREA scores with respect to patients’ characteristics

		18 items*	*p*-value	16 items*	p-value
		M (SD) / R		M (SD) / R	
Sex	Men	21.4 (13.6)	0.18	21.5 (13.,7)	0.355
	Women	22.6 (13.9)		22.5 (14.1)	
Age	<=65 years	22.9 (14.1)	0.017	23.1 (14.3)	0.007
	> 65 years	20.8 (13.3)		20.7 (13.4)	
Knaus score	Normal	22.9 (14.3)	0.192	23.3 (14.4)	0.090
	Moderate/severe	21.6 (13.6)		21.5 (13.7)	
MacCabe	Non fatal disease	22.5 (14.1)	0.080	22.6 (14.2)	0.045
	Fatal disease	20.9 (13.2)		20.8 (13.4)	
Type of admission	Medical	23.5 (14.6)	0.001	23.4 (14.7)	0.006
	Surgical	20.2 (12.6)		20.5 (12.8)	
SAPS II		0.099	0.002	0.088	0.006
Mechanical ventilation	No	21.6 (14.0)	0.417	21.6 (14.1)	0.416
	Yes	22.0 (13.5)		22.1 (13.7)	
Non mechanical ventilation	No	21.1 (13.4)	0.032	21.3 (13.6)	0.115
	Yes	23.3 (14.1)		23.0 (14.2)	
Vasopressors administration	No	21.7 (13.6)	0.203	21.8 (13.7)	0.146
	Yes	30.6 (20.8)		31.5 (20.8)	
Renal replacement therapy	No	21.3 (13.4)	0.132	21.4 (13.4)	0.170
	Yes	22.7 (14.3)		22.7 (14.4)	
ICU stay duration		0.231	0.001	0.219	0.132

*scores ranging from 0 to 100; the higher the score, the higher the discomfort

SAPS II Simplified Acute Physiology Score II (range from 0 to 156, with higher scores indicating more severe illness)

## Discussion

The initial version of IPREA was developed and validated from a large sample of unselected ICU patients by a working group that include ICU physicians, ICU nurses, and experts on patient-reported outcomes assessment. Use of IPREA in the clinical routine highlighted the absence from the assessment of two significant discomforts: feeling depressed and shortness of breath occurring during the ICU stay. These two items were added to this initial version, leading to a total of 18 items. In this study, we reported the validation of the 18-item version of IPREA.

Concerning the psychometric properties, the 18-item version met standards and showed equivalent metric properties in comparison with the 16-item version that could raise questions about the utility of a longer version. The addition of two items did not modify the unidimensional structure of the tool supported by component factor analyses and Rasch analysis. The pattern of item goodness-of-fit confirmed that all the items measure the same concept. The overall score probably reflected quite a broad range of patient experience (favorable and unfavorable) and may have failed to identify some significant areas of patient distress; but item scores allowed a detailed description by type of discomfort.

However, we showed that these two additional items in the IPREA questionnaire, shortness of breath and feeling depressed, will be very useful for future studies, as well as to test the efficacy of programs regarding the eventual association between the self-reported score of feeling of depressed on the day of ICU discharge and the prevalence of severe symptoms of depression in survivors of critical illness, as measured, for example with specific tools such as the Hospital and Anxiety Depression scale several months after ICU discharge.

As with the 16-item version, the 18-item version of IPREA showed high floor effects leading to a low potential discriminative power or a difficulty in bringing out differences between groups, for example. However, this observation is usual in “satisfaction-like” tools, due to the undesirable nature of the measured trait. Perception of a discomfort may be considered an undesirable perception.

The question of completion by the bedside nurses is legitimate. In the future, the patients could directly report their scores on a digital platform. However, this suggestion is not easy in real life. On the day of ICU discharge, most of patients are not well enough to be able to optimally use an electronic device. To be used in a pragmatic clinical routine, we think that relying on nurses remains appropriate. The method of data collection employed, in which patients completed the questionnaire just before being discharged, may have over- or under estimated the level of discomfort compared to questionnaires completed at home. Future studies should be conducted based on external observers and repeated administration of the IPREA after ICU discharge. The reproducibility of the 18-item version should be assessed in future studies.

The scores of perceived ICU-related discomforts are rather low. This phenomenon could be partially explained by the Hawthorne effect [27, 28], i.e., more efforts were made by the medical staff to reduce potential sources of discomfort because they knew that they were observed.

The question of the importance of the effect should be examined through future studies by the determination of the minimal clinically important difference in the IPREA score(s) [29].

Finally, International collaborations should be planned in the future to perform linguistic validation process. Providing multiple language versions of a questionnaire allows researchers to pool data from different countries in multinational studies, to compare scores between countries and to establish norms.

## Conclusion

The 18-item version of IPREA is easy to use and possesses satisfactory psychometric properties. The availability of a reliable and valid French questionnaire concerning self-perceived patient discomforts during an ICU stay enables feedback from patients and health care teams to be incorporated in a continuous quality health care improvement strategy. The use of the 18-item version of IPREA in comparison with the 16-item version should be recommended because of its potential to improve tailored programs for reducing two self-perceived discomforts, frequently reported by patients and acknowledged by healthcare teams in the ICU, shortness of breath and feeling depressed.

## Abbreviations

CFA: Confirmatory factor analysis
CFI: Comparative fit index
ICU: Intensive care unit
INFIT: Item goodness-of-fit statistics
IPREA: Inconforts des patients de reanimation
MV: Mechanical ventilation
NIV: Non Invasive ventilation
RMSEA: Root mean square error of approximation
RRT: Renal replacement therapy
SAPS II: Simplified acute physiology score
TMCP: Tailored multicomponent program
